# Supplementary material for: Factors associated with the age of the onset of diabetes in women aged 50 years or more: a population-based study
Source: BMJ Open. 2014 Nov 26;4(11):e004838. doi: 10.1136/bmjopen-2014-004838 (PMC4248098; doi:10.1136/bmjopen-2014-004838)
Supplement: Author's manuscript [file bmjopen-2014-004838.draft_revisions.pdf]

Factors associated with the age of the onset of diabetes in women aged 50 years or more: a population-based study

Journal:	*BMJ Open*
Manuscript ID:	bmjopen-2014-004838
Article Type:	Research
Date Submitted by the Author:	10-Jan-2014
Complete List of Authors:	Valadares, Ana; School of Medical Sciences, University of Campinas (UNICAMP), Department of Obstetrics and Gynecology<br>Machado, Vanessa; School of Medical Sciences, University of Campinas (UNICAMP), Department of Obstetrics and Gynecology<br>Costa-Paiva, Lúcia; School of Medical Sciences, University of Campinas (UNICAMP), Department of Obstetrics and Gynecology<br>Sousa, Maria Helena; Campinas Center for Research in Reproductive Health (CEMICAMP), UNICAMP, Statistics<br>Pinto-Neto, Aarão; School of Medical Sciences, University of Campinas (UNICAMP), Department of Obstetrics and Gynecology
**Primary Subject Heading**:	Epidemiology
Secondary Subject Heading:	Diabetes and endocrinology, Nutrition and metabolism, Public health
Keywords:	EPIDEMIOLOGY, GYNAECOLOGY, Risk management < HEALTH SERVICES ADMINISTRATION & MANAGEMENT, Diabetes & endocrinology < INTERNAL MEDICINE

SCHOLARONE™ Manuscripts

Factors associated with the age of the onset of diabetes in women aged 50 years or more: a population-based study

Running title: Factors associated with diabetes

Ana L. R. Valadares, MD, PhD ;[1] Vanessa S. S. Machado, MSc;[1] Lúcia S. Costa-Paiva, MD, PhD; [1] Maria H. de Sousa, PhD; [2] Aarão M. Pinto-Neto, MD, PhD [1]

[1.] Department of Obstetrics and Gynecology, School of Medical Sciences, State University of Campinas (UNICAMP), Campinas, SP, Brazil.

[2.] Department of Statistics, Campinas Center for Research in Reproductive Health (CEMICAMP), UNICAMP, Campinas, SP, Brazil.

Corresponding author:

Ana Lúcia Ribeiro Valadares

Department of Obstetrics and Gynecology

School of Medical Sciences, PO Box 6111

University of Campinas (UNICAMP)

13083-970 Campinas, SP, Brazil

Telephone and Fax: +55 19 3521 9306

E-mail: anarvaladares@gmail.com

For peer review only

Abstract

Objective: Investigate factors associated with the onset of diabetes in women with more than 49 years.

Design and Methods: Cross-sectional, population-based study using self-reports with 622 women. The dependent variable was the age of occurrence of diabetes using the life table method. Cox multiple regression models were adjusted to analyze the onset of diabetes according to predictor variables. Sociodemographic, clinical and behavioral factors were evaluated.

Results: Of the 622 women interviewed, 22.7% had diabetes. The mean age at onset was 56 years. The factors associated with the age of occurrence of diabetes were self-rated health (very good, good) (coefficient = -0.792; standard error of the coefficient = 0.215; p = 0.0001), more than two individuals living in the household (coefficient = 0.656, standard error of the coefficient = 0.223; p = 0.003), and body mass index (BMI) (kg/m^2) at 20-30 years of age (coefficient = 0.056, standard error of the coefficient = 0.023; p = 0.014).

Conclusions: Self-rated health considered good or very good was associated with a higher rate of survival without diabetes. Sharing a home with two or more other people and a weight increase at 20-30 years of age was associated with the onset of type 2 diabetes.

Key words: diabetes; menopause; self-rated health; obesity.

For peer review only

Strengths and limitations of this study

Strengths

- Knowledge based on epidemiological population study about factors associated with the onset of diabetes in women with 50 or more years.
- Information that BMI control in young adults, better self-rated health and improvement of socioeconomic conditions could prevent the onset of diabetes in the aging process.
- Observation that was no association between menopausal status and the onset of diabetes.

Limitations

- The presence of diabetes mellitus was determined on the basis of self-reported physician-diagnosed diabetes, and confirmation of this diagnosis was not made.

For peer review only

Introduction

Type 2 diabetes is a chronic, heterogeneous, progressive metabolic disease that is characterized by insulin resistance. The relevance of this condition lies in its high prevalence and incidence, the individual burden of disease in patients due to macro- and microvascular complications, and the associated costs to the healthcare system [1]. In women with diabetes, life expectancy was found to be 5.8 years shorter than in women without diabetes, irrespective of income [2].

The prevalence of diabetes has increased worldwide, reaching epidemic proportions in recent years, as a result of population aging and obesity [3,4]. It is estimated that in 2011, 8.6% of individuals in Central and South America had diabetes, and predictions suggest that this percentage will reach 10.1% by 2030 [5]. In Brazil, the prevalence of diabetes is 13.5% in individuals of 30-79 years of age [6] and 18.7% in women over 60 years of age [7]. Hospitalizations due to diabetes mellitus account for 9% of hospital spending within the Brazilian National Health System (*Sistema Único de Saúde* – SUS) (8). In general, the prevalence of diabetes increases with aging, particularly after 45-50 years of age and the presence of diabetes also constitutes an important risk factor for cardiovascular disease. Physical inactivity, an inadequate diet and an increase in the prevalence of obesity are factors held responsible for the global expansion of diabetes [5].

One of the most used methods for estimating the prevalence of common chronic diseases such as type 2 diabetes is a nationwide or regional population survey. Such surveys are usually restricted to self-reported data on diabetes;

For peer review only

however, specificity has been found to be high, with the data from these surveys correlating well with the actual occurrence of the disease [9,10]. Diabetes is at the center of behavioral problems, and psychological and social factors play a crucial role in its management [11]; therefore, it is important to know which factors contribute towards its onset.

The objective of this study was to investigate the factors most strongly associated with the age at onset of diabetes in women of fifty years of age or more in a population-based study conducted in Brazil.

Methods and procedures

Subjects

A cross-sectional, population-based study using data derived from self-reports was conducted between May 10 and October 31, 2011 in the city of Campinas, São Paulo, Brazil. Sixty-eight census sectors (the primary sampling units) of the city of Campinas, Brazil were randomly selected by simple random sampling or equal probability of selection. The selection procedure was performed according to a table of random numbers generated from a list supplied by the Brazilian Institute of Geography and Statistics (IBGE) and classified according to the sector identification number (ID__). Prior to selection, the number of women of 50 years of age or more living in each census sector (women eligible for the study) was determined. Sectors with fewer than 10 women in this age group were grouped together with the neighboring sector holding the subsequent ID number. Research assistants, guided by maps of each census area, went to the odd-numbered houses and verified whether there were any women of 50 years of age or more living there. If there were eligible women

For peer review only

residing at the address, they were invited to participate in the research project and if they agreed, a questionnaire was applied personally by interviewers trained at the Campinas Center for Research and the Control of Maternal and Child Diseases (CEMICAMP) until 10 eligible women had been interviewed in each sector. If it proved impossible to interview 10 women in any given sector using this methodology, work was then resumed in that sector by visiting the addresses not included at the first attempt (i.e. the even-numbered houses). A total of 622 women effectively participated in the present study, since 99 out of 721 invitations (13.7%) were declined.

Sample size

The target population consisted of all the female residents of Campinas, São Paulo, Brazil, who were 50 years of age or more in 2007. This made a total of 131,800 women. To calculate sample size, the prevalence of diabetes was estimated at 13.5% [6]. A type I error of 5% was defined, with a margin of error of 4% (the absolute difference between the proportion in the sample and that of the population), resulting in a sample size of 280 women. Taking into consideration a possible loss of 10% of the subjects, the minimum sample size was increased to 308 women. The final sample obtained consisted of 622 women of 50 years of age or more.

This study forms part of a larger project conducted to evaluate the health conditions of women of 50 years of age or more. The project was approved by the internal review board of CAISM/UNICAMP and was conducted in compliance with the current version of the Declaration of Helsinki and with

For peer review only

Resolution 196/96 of the Brazilian National Committee for Ethics in Research (CONEP) and its subsequent revisions.

Inclusion and exclusion criteria

Women of 50 years of age or more were eligible, while those with any factor that prevented the interview from taking place were excluded. Precluding factors included lack of cognitive ability to answer the questionnaire, prior commitments and incompatibility of schedules.

Instrument

The participants answered a structured, pre-tested questionnaire created on the basis of three preexisting questionnaires. Of these, two were Brazilian questionnaires, one of which was part of the SABE project on health, well-being and aging in Latin America and the Caribbean [7], while the other formed part of a population-based survey denominated VIGITEL 2008, conducted by the Brazilian Ministry of Health [12]. The third questionnaire was used in the "Women's Health and Aging Study", a nationwide study conducted in the US (13). The present questionnaire was divided into five sections: sociodemographic evaluation, health-related habits, self-perception of health, and evaluation of functional capacity and health-related problems.

Variables

The independent variables consisted of: age (in years), marital status, years of schooling, number of people living in the household, skin color, smoking and alcohol consumption, having private medical insurance, practice of

For peer review only

physical exercise, having stopped menstruating more than a year ago; physician's diagnosis of menopause, body mass index (BMI) at 20-30 years of age, current BMI, and self-perception of health.

The dependent variable was the age at onset of diabetes reported by women at the time of interview. This information was obtained by asking women if they had the disease and whether it was diagnosed by a physician. With a positive answer, the individual was then asked on the time since diagnosis and on treatment. Thus the presence of diabetes was further validated.

Data analysis

First, the age of the onset of diabetes in annual intervals, reported by women at the time of interview, was used to calculate the cumulative continuation rates (survival) without diabetes, using the life table method. If the woman has not experienced diabetes at the time of the interview, it was considered a censored data [14]. Next, Cox multiple regression models were adjusted to analyze the rates of the age of the onset of diabetes in accordance with various predictive variables: schooling (≤ 8 years / > 8 years); marital status (with a partner / no partner); skin color (white / other); number of people living in the household (≤ 2 / > 2); smoking (never smoked / past or current smoker); number of cigarettes smoked per day currently or in the past (≤ 15 / > 15); alcohol consumption (yes/ no); frequency of alcohol consumption (none or < 12 day/month/ other); having private medical insurance (yes / no); weekly practice of physical exercise (yes / no); frequency of physical exercise (≤ 2 days/week/ ≥ 3 days/week); BMI

For peer review only

(kg/m^2) at 20-30 years of age; self-rated health (very good, good / other); menopausal (yes / no).

All the women participated voluntarily in the study and signed an informed consent form. The study protocol was approved by the internal review board of the School of Medical Sciences, University of Campinas.

Results

The sociodemographic characteristics of the women in the study sample are shown in Table 1.

Of the 622 women interviewed, 22.7% reported having diabetes. Of the women with diabetes (n=140), the mean age at onset of the disease was 56 ± 11.2 years (median 55 years), reported at the time of interview (Figure 1). The factors associated with the age of occurrence of diabetes were self-rated health (very good, good) (coefficient = - 0.792, standard error of the coefficient = 0.215; p = 0.001), more than two people living in the household (coefficient = 0.656; standard error of the coefficient = 0.223; p = 0.003); and BMI (kg/m^2) at 20-30 years of age (coefficient = 0.056, standard error of the coefficient = 0.023; p = 0.014) (Table 2). No association was found between menopausal status and diabetes.

Discussion

The objective of this population-based study was to evaluate factors associated with the age at onset of diabetes in women above 49 years.

For peer review only

In the current study the prevalence of self-reported diabetes was 22.7%, which could lead to misreporting. This finding is consistent with other studies. In Brazil Lebrão et al. showed a 18.7% prevalence of self reported diabetes among women over 60 years of age [7], and in the USA, for the period of 2005–2008, it was estimated that 26.9% of people aged 65 or more had diabetes, based on both fasting glucose and A1C levels [15].

Self-rated health considered good or very good was associated with a higher rate of survival without diabetes. In line with this finding, a prospective case-cohort study reported that poor self-rated health was associated with a higher risk of type 2 diabetes [16]. Previous studies conducted with occupational cohorts have suggested that self-rated health principally indicates physical and mental health problems and, to a lesser extent, age, early life factors, family history, sociodemographic variables, psychosocial factors and health-related behavior [17,18].

Sharing a home with more than one person was associated with the presence of diabetes. Reports in the literature on the number of individuals sharing a home and the presence of diabetes are conflicting. In a population cohort that included both men and women, an association was found between living alone and type 2 diabetes in men; however, there was no increased risk for women living alone [19]. Nevertheless, a Swedish study investigating the role of household conditions in the progression from impaired glucose tolerance to diabetes in 461 women of 50 to 64 years of age found that women living alone had a 2.7-fold increased risk of type 2 diabetes even after adjustment for

For peer review only

biological risk factors [20]. In other countries, living alone is believed to be related to poor perceived social support, lack of a close confidant and poor emotional support, and may be a proxy for poor social support and consequently social isolation [21]. We may hypothesize that the difference between the findings of the present study and those of Lidfeldt et al. [20]may be explained by the fact that in Brazil the women most likely to have type 2 diabetes are older and share a home with other people because they require care. In addition, they may have lower incomes and poorer health conditions. A large body of evidence suggests that socioeconomically disadvantaged groups are at an increased risk of type 2 diabetes [22,23].

A BMI increase at 20-30 years of age was another factor associated with the onset of diabetes. In a longitudinal study enrolling adults over 35 years of age with no cardiovascular disease or diabetes conducted during a 7 year follow-up period, the BMI cut-off of 30kg/m^2 was associated with a 1.94-fold (1.42-2.66) increased risk of type 2 diabetes [24]. Jeffreys et al. also have demonstrated that overweight at any point in a person's life is associated with an increased risk of developing diabetes and the risk associated with being overweight is cumulative across the lifecourse [25].

No association was found between menopausal status and the onset of diabetes in the present study. In agreement with this finding, Kim et al. reported that in the women in the Diabetes Prevention Program who were at a high risk for diabetes, natural menopause did not increase this risk [26]. Nevertheless most

For peer review only

of the previous studies were small, hence insufficiently powered to answer this question [27].

Some limitations of this study must be taken into consideration. As in most population-based studies, the presence of diabetes mellitus was determined on the basis of self-reported physician-diagnosed diabetes, and confirmation of this diagnosis was not made. Nevertheless, the onset of a disease so important like diabetes is generally remembered which decreases the risk of remembering bias. Furthermore, in the present study the age of the occurrence of diabetes was also based in the report of the diagnosis made by the physician. The reliability of self-reported diabetes mellitus has been previously validated [2]. The fact of the study has a population-based nature represents an important strongpoint. The representativeness of the population sample allows these conclusions to be extrapolated to the entire population of women of 50 years of age or more in a Brazilian city. Population-based estimates of the age of occurrence of diabetes in women of fifty years of age or more and its associated factors are important for understanding this issue in women's lives as they age, while designing interventions in the field of diabetes prevention requires a good knowledge of region-specific trends.

These results contribute to highlight the need to target weight control interventions earlier in life and for measures aimed to improve women's socioeconomic conditions during the aging process to prevent type 2 diabetes.

For peer review only

Acknowledgements

Financial Support: The São Paulo Foundation for the Support of Research (FAPESP), grant # 04/10524-8.

Funding

This works was funded by The São Paulo Foundation for the Support of Research (FAPESP), grant # 04/10524-8.

Contributorship Statement

Conception and Design

Aarão Mendes Pinto-Neto, Vanessa S S Machado, Ana Lúcia Ribeiro Valadares

Acquisition of Data

Vanessa S S Machado; Aarão Mendes Pinto-Neto

Analysis and Interpretation of Data

Maria Helena Sousa ; Aarão Mendes Pinto-Neto; Ana Lúcia Ribeiro Valadares;

Drafting the Article

Ana Lúcia Ribeiro Valadares; Aarão Mendes Pinto-Neto, Lúcia Costa Paiva

Revising It for Intellectual Content

Ana Lúcia Ribeiro Valadares; Aarão Mendes Pinto-Neto; Lúcia Costa Paiva; Maria Helena Sousa; Vanessa SS Machado

Final Approval of the Completed Article

For peer review only

Ana Lúcia Ribeiro Valadares; Aarão Mendes Pinto-Neto; Lúcia Costa Paiva; Maria Helena Sousa; Vanessa SS Machado

Conflicts of interest statement

As corresponding author, I confirm that I have collected ICMJE Uniform Disclosure Forms for Potential Conflicts of Interest from every author and no Conflicts of Interest exist for any of the authors.

Data Sharing Statement

We don´t have any other unpublished data from the study on diabetes.

For peer review only

References

1. Mensing C, Boucher J, Cypress M, et al. National standards for diabetes self-management education. *Diabetes Care* 2005;28 Suppl 1:S72-79.
2. Emerging Risk Factors Collaboration, Seshasai SR, Kaptoge S, Thompson A, et al. Diabetes mellitus, fasting glucose, and risk of cause-specific death. *N Engl J Med* 2011;364:829-841.
3. Whiting DR, Guariguata L, Weil C, Shaw J. IDF diabetes atlas: global estimates of the prevalence of diabetes for 2011 and 2030. *Diabetes Res Clin Pract* 2011;94:311-321.
4. Pi-Sunyer FX. The obesity epidemic: pathophysiology and consequences of obesity. *Obes Res*;10 Suppl 2:97S-104S
5. Falagas ME, Vardakas KZ, Vergidis PI. Under-diagnosis of common chronic diseases: prevalence and impact on human health. *Int J Clin Pract* 2007;61:1569-1579.
6. Bosi PL, Carvalho AM, Contrera D, et al. Prevalence of diabetes and impaired glucose tolerance in the urban population of 30 to 79 years of the city of São Carlos, São Paulo]. *Arq Bras Endocrinol Metabol* 2009;53:726-732.
7. Lebrão ML, Duarte YAO. SABE – Saúde, bem-estar e envelhecimento. O projeto SABE no município de São Paulo: uma abordagem inicial. Brasília (DF): Organização Pan-Americana da Saúde, 2003. Available at: http://www.fsp.usp.br/sabe/Extras/Livro_SABE.pdf. Accessed April 20, 2011.

For peer review only

8. Rosa RS, Schmidt MI. Diabetes mellitus: magnitude das hospitalizações na rede pública do Brasil, 1999-2001. *Epidemiol Serv Saúde* 2008;17:123-153.

9. Köster I, Schubert I, Huppertz E. [Follow up of the CoDiM-Study: cost of diabetes mellitus 2000-2009]. *Dtsch Med Wochenschr* 2012;137:1013-1016.

10. Vargas CM, Burt VL, Gillum RF, Pamuk ER. Validity of self-reported hypertension in the National Health and Nutrition Examination Survey III, 1988-1991. *Prev Med* 1997;26(5 Pt 1):678-685.

11. Snoek FJ. *Management of diabetes: psychological aspects of diabetes management*. Abingdon: The Medicine Publishing Company, 2002.

12. Ministério da Saúde – Brasil. Vigilância de fatores de risco e proteção para doenças crônicas não transmissíveis por entrevistas telefônicas (VIGITEL). Available at: http://portal.saude.gov.br/portal/arquivos/pdf/167_Q2008.pdf. Accessed August 1st, 2011.

13. Guralnik JM, Fried LP, Simonsick EM, Kasper JD, Lafferty ME, eds. *The Women's Health and Aging Study: health and social characteristics of older women with disability*. NIH Pub. No. 95-4009. Bethesda, MD: National Institute on Aging, 1995.

14. Lee ET. Statistical methods for survival data analysis. Belmont. CA: Lifetime Learning Publications, 1980

15. National Diabetes Statistics, 2011. http://diabetes.niddk.nih.gov/dm/pubs/statistics/. Accessed August 1st, 2012.

For peer review only

16. Wennberg P, Rolandsson O, van der A DL, et al. Self-rated health and type 2 diabetes risk in the European Prospective Investigation into Cancer and Nutrition-InterAct study: a case-cohort study. *BMJ Open* 2013;3(3). pii: e002436. doi: 10.1136/bmjopen-2012-002436.
17. Singh-Manoux A, Martikainen P, Ferrie J, Zins M, Marmot M, Goldberg M. What does self rated health measure? Results from the British Whitehall II and French Gazel cohort studies. *J Epidemiol Community Health* 2006;60:364-372.
18. Machado VS, Valadares AL, da Costa-Paiva LS, de Souza MH, Osis MJ, Pinto-Neto AM. Factors associated with self-perception of health among Brazilian women 50 years or older: a population-based study. *Menopause* 2013;20:1055-60.
19. Meisinger C, Kandler U, Ladwig KH. Living alone is associated with an increased risk of type 2 diabetes mellitus in men but not women from the general population: the MONICA/KORA Augsburg Cohort Study. *Psychosom Med* 2009;71:784-788.
20. Lidfeldt J, Nerbrand C, Samsioe G, Agardh CD. Women living alone have an increased risk to develop diabetes, which is explained mainly by lifestyle factors. *Diabetes Care* 2005;28:2531-2536.
21. Schmaltz HN, Southern D, Ghali WA, et al. Living alone, patient sex and mortality after acute myocardial infarction. *J Gen Intern Med* 2007;22:572-578.
22. Agardh E, Allebeck P, Hallqvist J, Moradi T, Sidorchuk A. Type 2 diabetes incidence and socio-economic position: a systematic review and meta- analysis. *Int J Epidemiol* 2011;40:804-818.

For peer review only

23. Stringhini S, Tabak AG, Akbaraly TN, et al. Contribution of modifiable risk factors to social inequalities in type 2 diabetes: prospective Whitehall II cohort study. *BMJ* 2012;345:e5452.

24. Talaei M, Sadeghi M, Marshall T, et al. Anthropometric indices predicting incident type 2 diabetes in an Iranian population: The Isfahan Cohort Study. *Diabetes Metab* 2013 Jul 15. pii: S1262-3636(13)00080-3. doi: 10.1016/j.diabet.2013.04.001.

25. Jeffreys M, Lawlor DA , Galobardes B, McCarron P, Kinra S, Ebrahim S, Smith GD. Lifecourse weight patterns and adult-onset diabetes: the Glasgow Alumni and British Women's Heart and Health studies. *Int J of Obes* 2006; 30:507–512.

26. Kim C, Edelstein SL, Crandall JP, et al., Diabetes Prevention Program Research Group. Menopause and risk of diabetes in the Diabetes Prevention Program. *Menopause* 2011;18:857-868.

27. Morling JR, Balkau B, Wild SH. Diabetes in women - a life-course approach. *Menopause Int* 2013;19:87-95.

28. Rockwood K, Awalt E, MacKnight C, McDowell I. Incidence and outcomes of diabetes mellitus in elderly people: report from the Canadian Study of Health and Aging. *CMAJ* 2000;162:769-772.

For peer review only

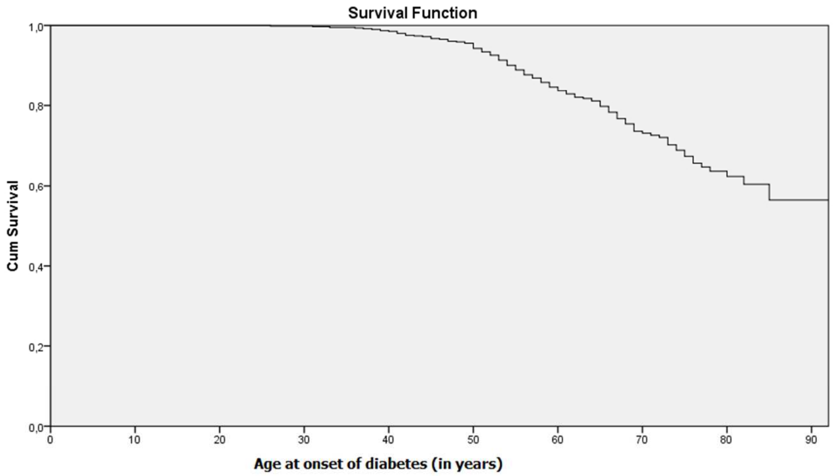

273x134mm (96 x 96 DPI)

For peer review only

Figure Legend

Figure 1: Age at the onset of diabetes over lifetime (years)

Cumulative survival

N=611

Mean age at onset of the disease was 56 ± 11.2 years (median 55 years)

Cumulative continuation rate without diabetes was 56% at 92 years of age

For peer review only

Table 1: Distribution of the women according to their sociodemographic and behavioral characteristics

Variable	(n)	%
Age (years)		
50 – 59	(234)	38.8
60 – 69	(193)	32.0
≥ 70	(176)	30.2
Schooling (years)		
≤ 8	(424)	70.3
> 8	(179)	29.7
Marital status		
No partner	(310)	51.4
Partner	(292)	48.6
Skin color/ethnicity		
White	(423)	71.0
Other	(172)	29.0
Monthly income (USD)		
≤ USD 750	(207)	53.2
> USD 750	(182)	46.8
Smoking		
Never smoked	(387)	64.2
Used to smoke	(140)	23.2
Smoker	(76)	12.6
Woman has private medical insurance		
Yes	(292)	48.4

For peer review only

No	(311)	51.6
Woman stopped menstruating over one year ago		
Yes	(564)	93.7
No	(38)	6.3
BMI (kg/m^2)		
<20.0	(26)	5.4
20.0 - 24.9	(155)	31.8
25.0 – 29.0	(181)	37.2
≥30	(125)	25.6
BMI (kg/m^2) at 20-30 years of age		
<20	(156)	36.1
20 -24.9	(222)	51.4
≥25	(54)	12.5

For peer review only

Table 2: Variables associated with the presence of Diabetes– Cox multiple regression analysis (n = 428)

Variable	Coefficient	Standard error of the coefficient	P-value
Self-rated health (very good, good)	-0.792	0.215	<0.001
Number of individuals living in the household (> 2)	0.656	0.223	0.003
BMI (kg/m^2) at 20–30 years of age	0.056	0.023	0.014

Predictor variables taken into consideration: schooling (≤ 8 years: 0 / > 8 years: 1); marital status (with a partner: 1 / no partner: 0); skin color (white: 1 / other: 0); number of people living in the household (≤ 2: 0 / > 2: 1); smoking (never smoked: 0 / past or current smoker: 1); number of cigarettes smoked per day currently or in the past (≤ 15: 0 / > 15: 1); alcohol consumption (yes: 1 / no: 0); frequency of alcohol consumption (none or < 1 day/month: 0 / other: 1); woman has private medical insurance (yes: 1 / no: 0); practices physical exercise weekly (yes: 1 / no: 0); frequency of physical exercise (≤ 2 days/week: 0 / ≥ 3 days/week: 1); BMI (kg/m^2) at 20-30 years of age; self-rated health (very good, good: 1 / other: 0); menopausal (yes: 1; no: 0).

For peer review only

UNICAMP **HEALTH CONDITIONS IN WOMEN OVER 50 YEARS OF AGE: A POPULATION-BASED STUDY CONDUCTED IN THE CITY OF CAMPINAS, SÃO PAULO, BRAZIL.**

Questionnaire

Questions refer to the manuscript: bmjopen-2014-004838

FACTORS ASSOCIATED WITH AGE AT THE ONSET OF DIABETES IN WOMEN OF 50 YEARS OF AGE OR MORE: A POPULATION-BASED STUDY

Study number: |___|___|___|

Census sector: ____________________

Interviewer: ____

Date: ___/___/___

SECTION 1 – SOCIODEMOGRAPHIC EVALUATION

I. Interviewer says: We will begin the questionnaire with some questions on your social status.

1.1 How old are you? |___|___| years [99] Did not answer

1.2 Up to what grade did you study?

_________ grade of ______________ Primary/Secondary School/College/University

[88] None [99] Did not answer

1.3 At this moment, are you single, married/living with a partner, separated/divorced or widowed?

[1] Single [4] Widowed

[2] Married/ Living with a partner [9] Did not answer

[3] Separated/Divorced

1.4 Regarding your ethnicity, are you: white, black, yellow, brown-skinned or indian?

[1] White [3] Yellow [5] Indian [9] Did not answer

[2] Black [4] Brown-skinned [8] Does not know

For peer review only

1.5 Including yourself, how many people live in your home? |___|___| persons

SECTION 2 – HEALTH HABITS

2. Interviewer says: I am now going to ask you some questions about your lifestyle habits.

2.1 Do you smoke, did you used to smoke or have you never smoked?
[1] Yes, I smoke [2] Yes, I used to smoke
[3] No, I have never smoked (go straight to 2.3).

2.2 How many cigarettes do you/did you smoke a day?
|___|___| Cigarettes [88] I don't know / don't remember

2.3 Do you have medical insurance?
[1] Yes [2] No [8] I don't know

2.4 Do you consume alcohol?
[1] Yes [2] No (Go straight to 2.14)

2.5 How often do you drink? (Read all the options before answering)
[1] Once or twice a week
[2] 3-4 days a week
[3] 5-6 days a week
[4] Every day
[5] Less than once a week
[6] Less than once a month

2.6 Do you practice physical exercise or participate in sports every week?
[1] Yes [2] No (Go straight to 2.16)

2.7 How often in a week do you practice physical exercise or participate in sports?
[1] Once a week [5] 5 times a week
[2] Twice a week [6] 6 times a week
[3] Three times a week [7] Every day
[4] 4 times a week

2.8 Has it been more than a year since you stopped menstruating?
[1] Yes [2] No (Go straight to 2.18)
[8] I don't remember (Go straight to 2.18)

For peer review only

2.9 How old were you when you stopped menstruating?

|___|___| Years [8] I don't know

2.10 Has a doctor ever told you that you are in the menopause?

[1] Yes [2] No [8] I don't remember

SECTION 3 – SELF-PERCEPTION OF HEALTH

3.1 How do you evaluate your health: (Read all the options before answering)

[1] Very good
[2] Good
[3] Average
[4] Poor
[5] Very poor

SECTION 4 – HEALTH PROBLEMS

4.1 How much did you weight when you were around 20-30 years of age?

|___|___|___|,|___| Kg [8] I don't know / don't remember

4.2 Has a doctor/healthcare professional ever diagnosed you with diabetes or high blood sugar levels?

[1] Yes [2] No [8] I don't know / don't remember

4.3 How long ago were you diagnosed with diabetes? (or the year in which you were diagnosed)

|___|___| years and/or |___|___| months [88] I don't know / don't remember

4.4 Are you treating your diabetes?

[1] Yes [2] No [8] I don't know / don't remember

For peer review only

BMJ Open

Factors associated with the age of the onset of diabetes in women aged 50 years or more: a population-based study

Journal:	*BMJ Open*
Manuscript ID:	bmjopen-2014-004838.R1
Article Type:	Research
Date Submitted by the Author:	n/a
Complete List of Authors:	Valadares, Ana; School of Medical Sciences, University of Campinas (UNICAMP), Department of Obstetrics and Gynecology<br>Machado, Vanessa; School of Medical Sciences, University of Campinas (UNICAMP), Department of Obstetrics and Gynecology<br>Costa-Paiva, Lúcia; School of Medical Sciences, University of Campinas (UNICAMP), Department of Obstetrics and Gynecology<br>Sousa, Maria Helena; Campinas Center for Research in Reproductive Health (CEMICAMP), UNICAMP, Statistics<br>Pinto-Neto, Aarão; School of Medical Sciences, University of Campinas (UNICAMP), Department of Obstetrics and Gynecology
**Primary Subject Heading**:	Epidemiology
Secondary Subject Heading:	Diabetes and endocrinology, Nutrition and metabolism, Public health
Keywords:	EPIDEMIOLOGY, GYNAECOLOGY, Risk management < HEALTH SERVICES ADMINISTRATION & MANAGEMENT, Diabetes & endocrinology < INTERNAL MEDICINE

SCHOLARONE™
Manuscripts

For peer review only

Factors associated with the age of the onset of diabetes in women aged 50 years or more: a population-based study

Running title: Factors associated with diabetes

Ana L. R. Valadares, MD, PhD ;[1] Vanessa S. S. Machado, MSc;[1] Lúcia S. Costa-Paiva, MD, PhD; [1] Maria H. de Sousa, PhD; [2] Aarão M. Pinto-Neto, MD, PhD [1]

[1.] Department of Obstetrics and Gynecology, School of Medical Sciences, State University of Campinas (UNICAMP), Campinas, SP, Brazil.

[2.] Department of Statistics, Campinas Center for Research in Reproductive Health (CEMICAMP), UNICAMP, Campinas, SP, Brazil.

Corresponding author:

Ana Lúcia Ribeiro Valadares

Department of Obstetrics and Gynecology

School of Medical Sciences, PO Box 6111

University of Campinas (UNICAMP)

13083-970 Campinas, SP, Brazil

Telephone and Fax: +55 19 3521 9306

E-mail: *anarvaladares@gmail.com*

For peer review only

Strengths and limitations of this study

Strengths

- Knowledge based on epidemiological population study about factors associated with the onset of diabetes in women with 50 or more years.
- Information that BMI control in young adults, better self-rated health and improvement of socioeconomic conditions could prevent the onset of diabetes in the aging process.
- Observation that was no association between menopausal status and the onset of diabetes.

Limitations

- The age of the occurrence of diabetes was also based on the report of the diagnosis made by the physician and other degrees of abnormal glucose tolerance were not taken into account.
- It was not possible to consider all factors that can impact on risks related to diabetes I like health problems, gestational diabetes and dietary intake or type of foods consumed.

Abstract

Objective: Investigate factors associated with the onset of diabetes in women with more than 49 years. *Design and Methods:* Cross-sectional, population-based study using self-reports with 622 women. The dependent variable was the age of occurrence of diabetes using the life table method. Cox multiple regression models were adjusted to analyze the onset of diabetes according to predictor variables. Sociodemographic, clinical and behavioral factors were evaluated. *Results:* Of the 622 women interviewed, 22.7% had diabetes. The mean age at onset was 56 years. The factors associated with the age of

For peer review only

occurrence of diabetes were self-rated health (very good, good) (coefficient = -0.792; standard error of the coefficient = 0.215; p = 0.0001), more than two individuals living in the household (coefficient = 0.656, standard error of the coefficient = 0.223; p = 0.003), and body mass index (BMI) (kg/m^2) at 20-30 years of age (coefficient = 0.056, standard error of the coefficient = 0.023; p = 0.014). *Conclusions:* Self-rated health considered good or very good was associated with a higher rate of survival without diabetes. Sharing a home with two or more other people and a weight increase at 20-30 years of age was associated with the onset of type 2 diabetes.

Key words: diabetes; menopause; self-rated health; obesity.

Introduction

Type 2 diabetes is a chronic, heterogeneous, progressive metabolic disease that is characterized by insulin resistance. The relevance of this condition lies in its high prevalence and incidence, the individual burden of disease in patients due to macro- and microvascular complications, and the associated costs to the healthcare system [1]. In women with diabetes, life expectancy was found to be 5.8 years shorter than in women without diabetes, irrespective of income [2].

The prevalence of diabetes has increased worldwide, reaching epidemic proportions in recent years, as a result of population aging and obesity [3,4,5]. It is estimated that in 2011, 8.6% of individuals in Central and South America had diabetes, and predictions suggest that this percentage will reach 10.1% by 2030. In Brazil, the prevalence of diabetes is 13.5% in individuals of 30-79 years

For peer review only

of age [6] and 18.7% in women over 60 years of age [7]. Hospitalizations due to diabetes mellitus account for 9% of hospital spending within the Brazilian National Health System (*Sistema Único de Saúde* – SUS) [8]. In general, the prevalence of diabetes increases with aging, particularly after 45-50 years of age and the presence of diabetes also constitutes an important risk factor for cardiovascular disease. Physical inactivity, an inadequate diet and an increase in the prevalence of obesity are factors held responsible for the global expansion of diabetes [5].

One of the most used methods for estimating the prevalence of common chronic diseases such as type 2 diabetes is a nationwide or regional population survey. Such surveys are usually restricted to self-reported data on diabetes; however, specificity has been found to be high, with the data from these surveys correlating well with the actual occurrence of the disease [9,10]. Diabetes is at the center of behavioral problems, and psychological and social factors play a crucial role in its management [11]; therefore, it is important to know which factors contribute towards its onset.

The objective of this study was to investigate the factors most strongly associated with the age at onset of diabetes in women of fifty years of age or more in a population-based study conducted in Brazil.

For peer review only

Methods and procedures

Subjects

A cross-sectional, population-based study using data derived from self-reports was conducted between May 10 and October 31, 2011 in the city of Campinas, São Paulo, Brazil. Sixty-eight census sectors (the primary sampling units) of

the city of Campinas, Brazil were randomly selected by simple random sampling or equal probability of selection. The selection procedure was performed according to a table of random numbers generated from a list supplied by the Brazilian Institute of Geography and Statistics (IBGE) and classified according to the sector identification number (ID__). Prior to selection, the number of women of 50 years of age or more living in each census sector (women eligible for the study) was determined. Sectors with fewer than 10 women in this age group were grouped together with the neighboring sector holding the subsequent ID number. Research assistants, guided by maps of each census area, went to the odd-numbered houses and verified whether there were any women of 50 years of age or more living there. If there were eligible women residing at the address, they were invited to participate in the research project and if they agreed, a questionnaire was applied personally by interviewers trained at the Campinas Center for Research and the Control of Maternal and Child Diseases (CEMICAMP) until 10 eligible women had been interviewed in each sector. If it proved impossible to interview 10 women in any given sector using this methodology, work was then resumed in that sector by visiting the addresses not included at the first attempt (i.e. the even-numbered houses). A total of 622 women effectively participated in the present study, since 99 out of 721 invitations (13.7%) were declined.

Sample size

The target population consisted of all the female residents of Campinas, São Paulo, Brazil, who were 50 years of age or more in 2007. This made a total of 131,800 women. To calculate sample size, the prevalence of diabetes was

For peer review only

estimated at 13.5% [6]. A type I error of 5% was defined, with a margin of error of 4% (the absolute difference between the proportion in the sample and that of the population), resulting in a sample size of 280 women. Taking into consideration a possible loss of 10% of the subjects, the minimum sample size was increased to 308 women. The final sample obtained consisted of 617 women of 50 years of age or more.

This study forms part of a larger project conducted to evaluate the health conditions of women of 50 years of age or more. The project was approved by the internal review board of CAISM/UNICAMP and was conducted in compliance with the current version of the Declaration of Helsinki and with Resolution 196/96 of the Brazilian National Committee for Ethics in Research (CONEP) and its subsequent revisions.

Inclusion and exclusion criteria

Women of 50 years of age or more were eligible, while those with any factor that prevented the interview from taking place were excluded. Precluding factors included lack of cognitive ability to answer the questionnaire, prior commitments and incompatibility of schedules.

Instrument

The participants answered a structured, pre-tested questionnaire created on the basis of three preexisting questionnaires. Of these, two were Brazilian questionnaires, one of which was part of the SABE project on health, well-being and aging in Latin America and the Caribbean [7], while the other formed part of a

For peer review only

population-based survey denominated VIGITEL 2008, conducted by the Brazilian Ministry of Health [12]. The third questionnaire was used in the "Women's Health and Aging Study", a nationwide study conducted in the US [13] The present questionnaire was divided into five sections: sociodemographic evaluation, health-related habits, self-perception of health, and evaluation of functional capacity and health-related problems.

Variables

The independent variables consisted of: age (in years), marital status, years of schooling, number of people living in the household, skin color, smoking and alcohol consumption, having private medical insurance, practice of physical exercise, having stopped menstruating more than a year ago; physician's diagnosis of menopause, body mass index (BMI) at 20-30 years of age, current BMI, and self-perception of health.

The dependent variable was the age at onset of diabetes reported by women at the time of interview. This information was obtained by asking women if they had the disease and whether it was diagnosed by a physician. With a positive answer, the individual was then asked on the time since diagnosis and on treatment. Thus the presence of diabetes was further validated.

Data analysis

First, the age of the onset of diabetes in annual intervals, reported by women at the time of interview, was used to calculate the cumulative continuation rates (survival) without diabetes, using the life table method. If the woman has not

For peer review only

experienced diabetes at the time of the interview, it was considered a censored data [14]. Next, Cox multiple regression models were adjusted to analyze the rates of the age of the onset of diabetes in accordance with various predictive variables: schooling (≤ 8 years / > 8 years); marital status (with a partner / no partner); skin color (white / other); number of people living in the household (≤ 2 / > 2); smoking (never smoked / past or current smoker); number of cigarettes smoked per day currently or in the past (≤ 15 / > 15); alcohol consumption (yes/ no); frequency of alcohol consumption (none or < 12 day/month/ other); having private medical insurance (yes / no); weekly practice of physical exercise (yes / no); frequency of physical exercise (≤ 2 days/week/ ≥ 3 days/week); BMI (kg/m^2) at 20-30 years of age; self-rated health (very good, good / other); menopausal (yes / no).

All the women participated voluntarily in the study and signed an informed consent form. The study protocol was approved by the internal review board of the School of Medical Sciences, University of Campinas.

Results

The sociodemographic characteristics of the women in the study sample are shown in Table 1.

Of the 617 women interviewed, 22.7% reported having diabetes. Of the women with diabetes (n=140), the mean age at onset of the disease was 56 ± 11.2 years (median 55 years), reported at the time of interview (Figure 1). The factors associated with the age of occurrence of diabetes were self-rated health (very good, good) (coefficient = - 0.792, standard error of the coefficient =

For peer review only

0.215; p = 0.001), more than two people living in the household (coefficient = 0.656; standard error of the coefficient = 0.223; p = 0.003); and BMI (kg/m^2) at 20-30 years of age (coefficient = 0.056, standard error of the coefficient = 0.023; p = 0.014) (Table 2). No association was found between menopausal status and diabetes.

Discussion

The objective of this population-based study was to evaluate factors associated with the age at onset of diabetes in women above 49 years.

In the current study the prevalence of self-reported diabetes was 22.7%, which could lead to misreporting. This finding is consistent with other studies. In Brazil Lebrão et al. showed a 18.7% prevalence of self reported diabetes among women over 60 years of age [7], and in the USA, for the period of 2005–2008, it was estimated that 26.9% of people aged 65 or more had diabetes, based on both fasting glucose and A1C levels [15].

Self-rated health considered good or very good was associated with a higher rate of survival without diabetes. In line with this finding, a prospective case-cohort study reported that poor self-rated health was associated with a higher risk of type 2 diabetes [16]. Previous studies conducted with occupational cohorts have suggested that self-rated health principally indicates physical and mental health problems and, to a lesser extent, age, early life factors, family history, sociodemographic variables, psychosocial factors and health-related behavior [17,18]. As it was a cross-sectional study, one can only say that there was an

For peer review only

association of onset of diabetes with self-rated health. So, it was not possible to demonstrate that a poor self-rated health was a causative factor or the effect of the onset of diabetes, due to the design of this study.

Sharing a home with more than one person was associated with the presence of diabetes. Reports in the literature on the number of individuals sharing a home and the presence of diabetes are conflicting. In a population cohort that included both men and women, an association was found between living alone and type 2 diabetes in men; however, there was no increased risk for women living alone [19]. Nevertheless, a Swedish study investigating the role of household conditions in the progression from impaired glucose tolerance to diabetes in 461 women of 50 to 64 years of age found that women living alone had a 2.7-fold increased risk of type 2 diabetes even after adjustment for biological risk factors [20]. In other countries, living alone is believed to be related to poor perceived social support, lack of a close confidant and poor emotional support, and may be a proxy for poor social support and consequently social isolation [21]. We may hypothesize that the difference between the findings of the present study and those of Lidfeldt et al. [20]may be explained by the fact that in Brazil the women most likely to have type 2 diabetes are older and share a home with other people because they require care. In addition, one may also hypothesize that these women may have lower incomes and poorer health conditions. A large body of evidence suggests that socioeconomically disadvantaged groups are at an increased risk of type 2 diabetes [22,23].

For peer review only

A BMI increase at 20-30 years of age was another factor associated with the onset of diabetes. Studies showed that being obese or overweight in a younger age may increase the risk of developing diabetes.[24,25] In a longitudinal study enrolling adults over 35 years of age with no cardiovascular disease or diabetes conducted during a 7 year follow-up period, the BMI cut-off of 30kg/m^2 was associated with a 1.94-fold (1.42-2.66) increased risk of type 2 diabetes [24]. Jeffreys et al. also have demonstrated that overweight at any point in a person's life is associated with an increased risk of developing diabetes and the risk associated with being overweight is cumulative across the lifecourse [25].

No association was found between menopausal status and the onset of diabetes in the present study. In agreement with this finding, Kim et al. reported that in the women in the Diabetes Prevention Program who were at a high risk for diabetes, natural menopause did not increase this risk [26]. Nevertheless most of the previous studies were small, hence insufficiently powered to answer this question [27]. One also has to consider that the absence of association between menopausal status and risk for diabetes may be due the majority of women were already post-menopausal at the time of onset of diabetes, in the present study.

Some limitations of this study must be taken into consideration. As in most population-based studies, the presence of diabetes mellitus was determined on the basis of self-reported physician-diagnosed diabetes, and confirmation of this diagnosis was not made. Nevertheless, the onset of a disease so important like diabetes is generally remembered which decreases the risk of remembering bias.[28] It was also not possible to consider all of the factors that can impact risk

For peer review only

of the onset of diabetes like health problems, gestational diabetes and dietary intake or type of foods consumed.[29]

Furthermore, in the present study the age of the occurrence of diabetes was also based in the report of the diagnosis made by the physician and other degrees of abnormal glucose tolerance were not taken in account. The reliability of self-reported diabetes mellitus has been previously validated [2]. The fact of the study has a population-based nature represents an important strongpoint. The representativeness of the population sample allows these conclusions to be extrapolated to the entire population of women of 50 years of age or more in a Brazilian city. Population-based estimates of the age of occurrence of diabetes in women of fifty years of age or more and its associated factors are important for understanding this issue in women's lives as they age, while designing interventions in the field of diabetes prevention requires a good knowledge of region-specific trends.

Conclusions

Self-rated health considered good or very good was associated with a higher rate of survival without diabetes. Sharing a home with two or more other people and a weight increase at 20-30 years of age was associated with the onset of type 2 diabetes.

These results contribute to highlight the need to target weight control interventions earlier in life and for measures aimed to improve women's socioeconomic conditions during the aging process to prevent type 2 diabetes.

For peer review only

a. Contributorship statement

Conception and Design

Aarão Mendes Pinto-Neto, Vanessa S S Machado, Ana Lúcia Ribeiro Valadares

Acquisition of Data

Vanessa S S Machado; Aarão Mendes Pinto-Neto

Analysis and Interpretation of Data

Maria Helena Sousa ; Aarão Mendes Pinto-Neto; Ana Lúcia Ribeiro Valadares;

Drafting the Article

Ana Lúcia Ribeiro Valadares; Aarão Mendes Pinto-Neto, Lúcia Costa Paiva

Revising It for Intellectual Content

Ana Lúcia Ribeiro Valadares; Aarão Mendes Pinto-Neto; Lúcia Costa Paiva; Maria Helena Sousa; Vanessa SS Machado

Final Approval of the Completed Article

Ana Lúcia Ribeiro Valadares; Aarão Mendes Pinto-Neto; Lúcia Costa Paiva; Maria Helena Sousa; Vanessa SS Machado

b. Competing interests

For peer review only

As corresponding author, I confirm that I have collected ICMJE Uniform Disclosure Forms for Potential Conflicts of Interest from every author and no Conflicts of Interest exist for any of the authors.

c. Funding

The São Paulo Foundation for the Support of Research (FAPESP), grant # 04/10524-8.

d. Data sharing

We confirm that is no additional unpublished data from the present study available.

Figure Legend

Figure 1: Age at the onset of diabetes over lifetime (years)

Cumulative survival

N=617

Mean age at onset of the disease was 56 ± 11.2 years (median 55 years)

Cumulative continuation rate without diabetes was 56% at 92 years of age

For peer review only

References

1. Mensing C, Boucher J, Cypress M, et al. National standards for diabetes self-management education. *Diabetes Care* 2005;28 Suppl 1:S72-79.
2. Emerging Risk Factors Collaboration, Seshasai SR, Kaptoge S, Thompson A, et al. Diabetes mellitus, fasting glucose, and risk of cause-specific death. *N Engl J Med* 2011;364:829-841.
3. Whiting DR, Guariguata L, Weil C, Shaw J. IDF diabetes atlas: global estimates of the prevalence of diabetes for 2011 and 2030. *Diabetes Res Clin Pract* 2011;94:311-321.
4. Pi-Sunyer FX. The obesity epidemic: pathophysiology and consequences of obesity. *Obes Res*;10 Suppl 2:97S-104S
5. Falagas ME, Vardakas KZ, Vergidis PI. Under-diagnosis of common chronic diseases: prevalence and impact on human health. *Int J Clin Pract* 2007;61:1569-1579.
6. Bosi PL, Carvalho AM, Contrera D, et al. Prevalence of diabetes and impaired glucose tolerance in the urban population of 30 to 79 years of the city of São Carlos, São Paulo]. *Arq Bras Endocrinol Metabol* 2009;53:726-732.
7. Lebrão ML, Duarte YAO. SABE – Saúde, bem-estar e envelhecimento. O projeto SABE no município de São Paulo: uma abordagem inicial. Brasília (DF): Organização Pan-Americana da Saúde, 2003. Available at: http://www.fsp.usp.br/sabe/Extras/Livro_SABE.pdf. Accessed April 20, 2011.

For peer review only

8. Rosa RS, Schmidt MI. Diabetes mellitus: magnitude das hospitalizações na rede pública do Brasil, 1999-2001. *Epidemiol Serv Saúde* 2008;17:123-153.

9. Köster I, Schubert I, Huppertz E. [Follow up of the CoDiM-Study: cost of diabetes mellitus 2000-2009]. *Dtsch Med Wochenschr* 2012;137:1013-1016.

10. Vargas CM, Burt VL, Gillum RF, et al. Validity of self-reported hypertension in the National Health and Nutrition Examination Survey III, 1988-1991. *Prev Med* 1997;26(5 Pt 1):678-685.

11. Snoek FJ. *Management of diabetes: psychological aspects of diabetes management*. Abingdon: The Medicine Publishing Company, 2002.

12. Ministério da Saúde – Brasil. Vigilância de fatores de risco e proteção para doenças crônicas não transmissíveis por entrevistas telefônicas (VIGITEL). Available at: http://portal.saude.gov.br/portal/arquivos/pdf/167_Q2008.pdf. Accessed August 1st, 2011.

13. Guralnik JM, Fried LP, Simonsick EM, et al. *The Women's Health and Aging Study: health and social characteristics of older women with disability*. NIH Pub. No. 95-4009. Bethesda, MD: National Institute on Aging, 1995.

14. Lee ET. Statistical methods for survival data analysis. Belmont. CA: Lifetime Learning Publications, 1980

15. National Diabetes Statistics, 2011. http://diabetes.niddk.nih.gov/dm/pubs/statistics/. Accessed August 1st, 2012.

For peer review only

16. Wennberg P, Rolandsson O, van der A DL, et al. Self-rated health and type 2 diabetes risk in the European Prospective Investigation into Cancer and Nutrition-InterAct study: a case-cohort study. *BMJ Open* 2013;3(3). pii: e002436. doi: 10.1136/bmjopen-2012-002436.
17. Singh-Manoux A, Martikainen P, Ferrie J, et al. What does self rated health measure? Results from the British Whitehall II and French Gazel cohort studies. *J Epidemiol Community Health* 2006;60:364-372.
18. Machado VS, Valadares AL, da Costa-Paiva LS, et al. Factors associated with self-perception of health among Brazilian women 50 years or older: a population-based study. *Menopause* 2013;20:1055-60.
19. Meisinger C, Kandler U, Ladwig KH. Living alone is associated with an increased risk of type 2 diabetes mellitus in men but not women from the general population: the MONICA/KORA Augsburg Cohort Study. *Psychosom Med* 2009;71:784-788.
20. Lidfeldt J, Nerbrand C, Samsioe G, et al. Women living alone have an increased risk to develop diabetes, which is explained mainly by lifestyle factors. *Diabetes Care* 2005;28:2531-2536.
21. Schmaltz HN, Southern D, Ghali WA, et al. Living alone, patient sex and mortality after acute myocardial infarction. *J Gen Intern Med* 2007;22:572-578.
22. Agardh E, Allebeck P, Hallqvist J, et al. Type 2 diabetes incidence and socio-economic position: a systematic review and meta- analysis. *Int J Epidemiol* 2011;40:804-818.

For peer review only

23. Stringhini S, Tabak AG, Akbaraly TN, et al. Contribution of modifiable risk factors to social inequalities in type 2 diabetes: prospective Whitehall II cohort study. *BMJ* 2012;345:e5452.

24. Talaei M, Sadeghi M, Marshall T, et al. Anthropometric indices predicting incident type 2 diabetes in an Iranian population: The Isfahan Cohort Study. *Diabetes Metab* 2013 Jul 15. pii: S1262-3636(13)00080-3. doi: 10.1016/j.diabet.2013.04.001.

25. Jeffreys M, Lawlor DA , Galobardes B, et al. Lifecourse weight patterns and adult-onset diabetes: the Glasgow Alumni and British Women's Heart and Health studies. *Int J of Obes* 2006; 30:507–512.

26. Kim C, Edelstein SL, Crandall JP, et al., Diabetes Prevention Program Research Group. Menopause and risk of diabetes in the Diabetes Prevention Program. *Menopause* 2011;18:857-868.

27. Morling JR, Balkau B, Wild SH. Diabetes in women - a life-course approach. *Menopause Int* 2013;19:87-95.

28. Rockwood K, Awalt E, MacKnight C, et al. Incidence and outcomes of diabetes mellitus in elderly people: report from the Canadian Study of Health and Aging. *CMAJ* 2000;162:769-772.

29. Bakhshi E, Seifi B, Biglarian A, et al. Changes in Body Mass Index across Age Groups in Iranian Women: Results from the National Health Survey.J Nutr Metab. 2012;2012:848403.

Table 1: Percentage of women without and with diabetes according to their sociodemographic and behavioral characteristics- Bivariate analysis

For peer review only

Variable	N	Without Diabetes %	With Diabetes %	p- value[2]
Age (years)	617			0.04
50 – 59		41.7	30.0	
60 – 69		30.8	36.4	
≥ 70		25.5	33.6	
Schooling (years)	617			0.20
≤ 8		67.9	78.6	
> 8		32.1	21.4	
Marital status[1]	609			0.59
Partner		48.7	45.7	
No partner		51.3	54.3	
Skin color/ethnicity[1]	609			0.76
Other		29.0	30.7	
White		71.0	69.3	
Monthly income (USD)[1]	397			0.07
≤ USD 750		50.8	62.2	
> USD 750		49.2	37.8	
Smoking	617			0.22
Never smoked		62.5	68.5	
Used to smoke		37.5	31.4	
Smoker				
number of cigarettes smoked per day currently or in the past	601			0.92
≤15		85.4	84.6	
>15		14.6	15.4	

For peer review only

Alcohol consumption	617			0.97
No		85.1	85.0	
Yes		14.9	15.0	
frequency of alcohol consumption	617			0.92
none or < 1 day/month		88.9	88.6	
> 1 day/month		11.1	11.4	
Woman has private medical insurance	617			0.17
No		50.1	57.1	
Yes		49.9	42.9	
Practices physical exercise weekly	617			0.16
No		62.3	69.3	
Yes		37.7	30.7	
Frequency of physical exercise[1]	616			0.74
Up to 2 days/week		73.1	75.0	
≥3 days/week		26.9	25.0	
BMI (kg/m^2) [1]	497			0.007
<25		40.3	25.5	
25 -29.9		36.7	40.0	
≥30		23.0	34.5	
BMI (kg/m^2) at 20-30 years of age[1]	440			0.001
<25		37.2	32.2	
25 -29.9		53.3	44.1	
≥30		9.5	23.7	
Self-rated health	617			0.0001
Other		36.7	56.4	

For peer review only

very good, good		63.3	43.6	
Woman stopped menstruating over one year ago[1]	615			0.95
No		13.4	13.7	
Yes		85.6	85.3	

[1]Some values may not add up to 617 because of missing data.

[2]X^2 test with Yates' correction.

Table 2: Variables associated with the presence of Diabetes– Cox multiple regression analysis (n = 428)

Variable	Coefficient	Standard error of the coefficient	P-value
Self-rated health (very good, good)	-0.792	0.215	<0.001
Number of individuals living in the household (> 2)	0.656	0.223	0.003
BMI (kg/m^2) at 20–30 years of age	0.056	0.023	0.014

Predictor variables taken into consideration: schooling (≤ 8 years: 0 / > 8 years: 1); marital status (with a partner: 1 / no partner: 0); skin color (white: 1 / other: 0); number of people living in the household (≤ 2: 0 / > 2: 1); smoking (never smoked: 0 / past or

For peer review only

current smoker: 1); number of cigarettes smoked per day currently or in the past (≤ 15: 0 / > 15: 1); alcohol consumption (yes: 1 / no: 0); frequency of alcohol consumption (none or < 1 day/month: 0 / other: 1); woman has private medical insurance (yes: 1 / no: 0); practices physical exercise weekly (yes: 1 / no: 0); frequency of physical exercise (≤ 2 days/week: 0 / ≥ 3 days/week: 1); BMI (kg/m^2) at 20-30 years of age; self-rated health (very good, good: 1 / other: 0); menopausal (yes: 1; no: 0).

For peer review only

Factors associated with the age of the onset of diabetes in women aged 50 years or more: a population-based study

Running title: Factors associated with diabetes

Ana L. R. Valadares, MD, PhD ;[1] Vanessa S. S. Machado, MSc;[1] Lúcia S. Costa-Paiva, MD, PhD; [1] Maria H. de Sousa, PhD; [2] Aarão M. Pinto-Neto, MD, PhD [1]

[1.] Department of Obstetrics and Gynecology, School of Medical Sciences, State University of Campinas (UNICAMP), Campinas, SP, Brazil.

[2.] Department of Statistics, Campinas Center for Research in Reproductive Health (CEMICAMP), UNICAMP, Campinas, SP, Brazil.

Corresponding author:

Ana Lúcia Ribeiro Valadares

Department of Obstetrics and Gynecology

School of Medical Sciences, PO Box 6111

University of Campinas (UNICAMP)

13083-970 Campinas, SP, Brazil

Telephone and Fax: +55 19 3521 9306

E-mail: *anarvaladares@gmail.com*

For peer review only

Strengths and limitations of this study

Strengths

- Knowledge based on epidemiological population study about factors associated with the onset of diabetes in women with 50 or more years.
- Information that BMI control in young adults, better self-rated health and improvement of socioeconomic conditions could prevent the onset of diabetes in the aging process.
- Observation that was no association between menopausal status and the onset of diabetes.

Limitations

- The age of the occurrence of diabetes was also based on the report of the diagnosis made by the physician and other degrees of abnormal glucose tolerance were not taken into account.
- It was not possible to consider all factors that can impact on risks related to diabetes l like health problems, gestational diabetes and dietary intake or type of foods consumed.

Abstract

Objective: Investigate factors associated with the onset of diabetes in women with more than 49 years. *Design and Methods:* Cross-sectional, population-based study using self-reports with 622 women. The dependent variable was the age of occurrence of diabetes using the life table method. Cox multiple regression models were adjusted to analyze the onset of diabetes according to predictor variables. Sociodemographic, clinical and behavioral factors were evaluated. *Results:* Of the 622 women interviewed, 22.7% had diabetes. The mean age at onset was 56 years. The factors associated with the age of

For peer review only

occurrence of diabetes were self-rated health (very good, good) (coefficient = -0.792; standard error of the coefficient = 0.215; p = 0.0001), more than two individuals living in the household (coefficient = 0.656, standard error of the coefficient = 0.223; p = 0.003), and body mass index (BMI) (kg/m^2) at 20-30 years of age (coefficient = 0.056, standard error of the coefficient = 0.023; p = 0.014). *Conclusions:* Self-rated health considered good or very good was associated with a higher rate of survival without diabetes. Sharing a home with two or more other people and a weight increase at 20-30 years of age was associated with the onset of type 2 diabetes.

Key words: diabetes; menopause; self-rated health; obesity.

Introduction

Type 2 diabetes is a chronic, heterogeneous, progressive metabolic disease that is characterized by insulin resistance. The relevance of this condition lies in its high prevalence and incidence, the individual burden of disease in patients due to macro- and microvascular complications, and the associated costs to the healthcare system [1]. In women with diabetes, life expectancy was found to be 5.8 years shorter than in women without diabetes, irrespective of income [2].

The prevalence of diabetes has increased worldwide, reaching epidemic proportions in recent years, as a result of population aging and obesity [3,4,5]. It is estimated that in 2011, 8.6% of individuals in Central and South America had diabetes, and predictions suggest that this percentage will reach 10.1% by 2030. In Brazil, the prevalence of diabetes is 13.5% in individuals of 30-79 years

For peer review only

of age [6] and 18.7% in women over 60 years of age [7]. Hospitalizations due to diabetes mellitus account for 9% of hospital spending within the Brazilian National Health System (*Sistema Único de Saúde* – SUS) [8]. In general, the prevalence of diabetes increases with aging, particularly after 45-50 years of age and the presence of diabetes also constitutes an important risk factor for cardiovascular disease. Physical inactivity, an inadequate diet and an increase in the prevalence of obesity are factors held responsible for the global expansion of diabetes [5].

One of the most used methods for estimating the prevalence of common chronic diseases such as type 2 diabetes is a nationwide or regional population survey. Such surveys are usually restricted to self-reported data on diabetes; however, specificity has been found to be high, with the data from these surveys correlating well with the actual occurrence of the disease [9,10]. Diabetes is at the center of behavioral problems, and psychological and social factors play a crucial role in its management [11]; therefore, it is important to know which factors contribute towards its onset.

The objective of this study was to investigate the factors most strongly associated with the age at onset of diabetes in women of fifty years of age or more in a population-based study conducted in Brazil.

Methods and procedures

Subjects

A cross-sectional, population-based study using data derived from self-reports was conducted between May 10 and October 31, 2011 in the city of Campinas, São Paulo, Brazil. Sixty-eight census sectors (the primary sampling units) of

For peer review only

the city of Campinas, Brazil were randomly selected by simple random sampling or equal probability of selection. The selection procedure was performed according to a table of random numbers generated from a list supplied by the Brazilian Institute of Geography and Statistics (IBGE) and classified according to the sector identification number (ID__). Prior to selection, the number of women of 50 years of age or more living in each census sector (women eligible for the study) was determined. Sectors with fewer than 10 women in this age group were grouped together with the neighboring sector holding the subsequent ID number. Research assistants, guided by maps of each census area, went to the odd-numbered houses and verified whether there were any women of 50 years of age or more living there. If there were eligible women residing at the address, they were invited to participate in the research project and if they agreed, a questionnaire was applied personally by interviewers trained at the Campinas Center for Research and the Control of Maternal and Child Diseases (CEMICAMP) until 10 eligible women had been interviewed in each sector. If it proved impossible to interview 10 women in any given sector using this methodology, work was then resumed in that sector by visiting the addresses not included at the first attempt (i.e. the even-numbered houses). A total of 622 women effectively participated in the present study, since 99 out of 721 invitations (13.7%) were declined.

Sample size

The target population consisted of all the female residents of Campinas, São Paulo, Brazil, who were 50 years of age or more in 2007. This made a total of 131,800 women. To calculate sample size, the prevalence of diabetes was

For peer review only

6

estimated at 13.5% [6]. A type I error of 5% was defined, with a margin of error of 4% (the absolute difference between the proportion in the sample and that of the population), resulting in a sample size of 280 women. Taking into consideration a possible loss of 10% of the subjects, the minimum sample size was increased to 308 women. The final sample obtained consisted of 617 women of 50 years of age or more.

This study forms part of a larger project conducted to evaluate the health conditions of women of 50 years of age or more. The project was approved by the internal review board of CAISM/UNICAMP and was conducted in compliance with the current version of the Declaration of Helsinki and with Resolution 196/96 of the Brazilian National Committee for Ethics in Research (CONEP) and its subsequent revisions.

Inclusion and exclusion criteria

Women of 50 years of age or more were eligible, while those with any factor that prevented the interview from taking place were excluded. Precluding factors included lack of cognitive ability to answer the questionnaire, prior commitments and incompatibility of schedules.

Instrument

The participants answered a structured, pre-tested questionnaire created on the basis of three preexisting questionnaires. Of these, two were Brazilian questionnaires, one of which was part of the SABE project on health, well-being and aging in Latin America and the Caribbean [7], while the other formed part of a

For peer review only

population-based survey denominated VIGITEL 2008, conducted by the Brazilian Ministry of Health [12]. The third questionnaire was used in the "Women's Health and Aging Study", a nationwide study conducted in the US (13). The present questionnaire was divided into five sections: sociodemographic evaluation, health-related habits, self-perception of health, and evaluation of functional capacity and health-related problems.

Variables

The independent variables consisted of: age (in years), marital status, years of schooling, number of people living in the household, skin color, smoking and alcohol consumption, having private medical insurance, practice of physical exercise, having stopped menstruating more than a year ago; physician's diagnosis of menopause, body mass index (BMI) at 20-30 years of age, current BMI, and self-perception of health.

The dependent variable was the age at onset of diabetes reported by women at the time of interview. This information was obtained by asking women if they had the disease and whether it was diagnosed by a physician. With a positive answer, the individual was then asked on the time since diagnosis and on treatment. Thus the presence of diabetes was further validated.

Data analysis

First, the age of the onset of diabetes in annual intervals, reported by women at the time of interview, was used to calculate the cumulative continuation rates (survival) without diabetes, using the life table method. If the woman has not

For peer review only

experienced diabetes at the time of the interview, it was considered a censored data [14]. Next, Cox multiple regression models were adjusted to analyze the rates of the age of the onset of diabetes in accordance with various predictive variables: schooling (≤ 8 years / > 8 years); marital status (with a partner / no partner); skin color (white / other); number of people living in the household (≤ 2 / > 2); smoking (never smoked / past or current smoker); number of cigarettes smoked per day currently or in the past (≤ 15 / > 15); alcohol consumption (yes/ no); frequency of alcohol consumption (none or < 12 day/month/ other); having private medical insurance (yes / no); weekly practice of physical exercise (yes / no); frequency of physical exercise (≤ 2 days/week/ ≥ 3 days/week); BMI (kg/m^2) at 20-30 years of age; self-rated health (very good, good / other); menopausal (yes / no).

All the women participated voluntarily in the study and signed an informed consent form. The study protocol was approved by the internal review board of the School of Medical Sciences, University of Campinas.

Results

The sociodemographic characteristics of the women in the study sample are shown in Table 1.

Of the 617 women interviewed, 22.7% reported having diabetes. Of the women with diabetes (n=140), the mean age at onset of the disease was 56 ± 11.2 years (median 55 years), reported at the time of interview (Figure 1). The factors associated with the age of occurrence of diabetes were self-rated health (very good, good) (coefficient = - 0.792, standard error of the coefficient =

For peer review only

0.215; p = 0.001), more than two people living in the household (coefficient = 0.656; standard error of the coefficient = 0.223; p = 0.003); and BMI (kg/m^2) at 20-30 years of age (coefficient = 0.056, standard error of the coefficient = 0.023; p = 0.014) (Table 2). No association was found between menopausal status and diabetes.

Discussion

The objective of this population-based study was to evaluate factors associated with the age at onset of diabetes in women above 49 years.

In the current study the prevalence of self-reported diabetes was 22.7%, which could lead to misreporting. This finding is consistent with other studies. In Brazil Lebrão et al. showed a 18.7% prevalence of self reported diabetes among women over 60 years of age [7], and in the USA, for the period of 2005–2008, it was estimated that 26.9% of people aged 65 or more had diabetes, based on both fasting glucose and A1C levels [15].

Self-rated health considered good or very good was associated with a higher rate of survival without diabetes. In line with this finding, a prospective case-cohort study reported that poor self-rated health was associated with a higher risk of type 2 diabetes [16]. Previous studies conducted with occupational cohorts have suggested that self-rated health principally indicates physical and mental health problems and, to a lesser extent, age, early life factors, family history, sociodemographic variables, psychosocial factors and health-related behavior [17,18]. As it was a cross-sectional study, one can only say that there was an

For peer review only

10

association of onset of diabetes with self-rated health. So, it was not possible to demonstrate that a poor self-rated health was a causative factor or the effect of the onset of diabetes, due to the design of this study.

Sharing a home with more than one person was associated with the presence of diabetes. Reports in the literature on the number of individuals sharing a home and the presence of diabetes are conflicting. In a population cohort that included both men and women, an association was found between living alone and type 2 diabetes in men; however, there was no increased risk for women living alone [19]. Nevertheless, a Swedish study investigating the role of household conditions in the progression from impaired glucose tolerance to diabetes in 461 women of 50 to 64 years of age found that women living alone had a 2.7-fold increased risk of type 2 diabetes even after adjustment for biological risk factors [20]. In other countries, living alone is believed to be related to poor perceived social support, lack of a close confidant and poor emotional support, and may be a proxy for poor social support and consequently social isolation [21]. We may hypothesize that the difference between the findings of the present study and those of Lidfeldt et al. [20]may be explained by the fact that in Brazil the women most likely to have type 2 diabetes are older and share a home with other people because they require care. In addition, one may also hypothesize that these women may have lower incomes and poorer health conditions. A large body of evidence suggests that socioeconomically disadvantaged groups are at an increased risk of type 2 diabetes [22,23].

For peer review only

A BMI increase at 20-30 years of age was another factor associated with the onset of diabetes. Studies showed that being obese or overweight in a younger age may increase the risk of developing diabetes.[24,25] In a longitudinal study enrolling adults over 35 years of age with no cardiovascular disease or diabetes conducted during a 7 year follow-up period, the BMI cut-off of 30kg/m^2 was associated with a 1.94-fold (1.42-2.66) increased risk of type 2 diabetes [24]. Jeffreys et al. also have demonstrated that overweight at any point in a person's life is associated with an increased risk of developing diabetes and the risk associated with being overweight is cumulative across the lifecourse [25].

No association was found between menopausal status and the onset of diabetes in the present study. In agreement with this finding, Kim et al. reported that in the women in the Diabetes Prevention Program who were at a high risk for diabetes, natural menopause did not increase this risk [26]. Nevertheless most of the previous studies were small, hence insufficiently powered to answer this question [27]. One also has to consider that the absence of association between menopausal status and risk for diabetes may be due the majority of women were already post-menopausal at the time of onset of diabetes, in the present study.

Some limitations of this study must be taken into consideration. As in most population-based studies, the presence of diabetes mellitus was determined on the basis of self-reported physician-diagnosed diabetes, and confirmation of this diagnosis was not made. Nevertheless, the onset of a disease so important like diabetes is generally remembered which decreases the risk of remembering bias.[28] It was also not possible to consider all of the factors that can impact risk

For peer review only

of the onset of diabetes like health problems, gestational diabetes and dietary intake or type of foods consumed.[29]

Furthermore, in the present study the age of the occurrence of diabetes was also based in the report of the diagnosis made by the physician and other degrees of abnormal glucose tolerance were not taken in account. The reliability of self-reported diabetes mellitus has been previously validated [2]. The fact of the study has a population-based nature represents an important strongpoint. The representativeness of the population sample allows these conclusions to be extrapolated to the entire population of women of 50 years of age or more in a Brazilian city. Population-based estimates of the age of occurrence of diabetes in women of fifty years of age or more and its associated factors are important for understanding this issue in women's lives as they age, while designing interventions in the field of diabetes prevention requires a good knowledge of region-specific trends.

Conclusions

Self-rated health considered good or very good was associated with a higher rate of survival without diabetes. Sharing a home with two or more other people and a weight increase at 20-30 years of age was associated with the onset of type 2 diabetes.

These results contribute to highlight the need to target weight control interventions earlier in life and for measures aimed to improve women's socioeconomic conditions during the aging process to prevent type 2 diabetes.

For peer review only

13

a. Contributorship statement

Conception and Design

Aarão Mendes Pinto-Neto, Vanessa S S Machado, Ana Lúcia Ribeiro Valadares

Acquisition of Data

Vanessa S S Machado; Aarão Mendes Pinto-Neto

Analysis and Interpretation of Data

Maria Helena Sousa ; Aarão Mendes Pinto-Neto; Ana Lúcia Ribeiro Valadares;

Drafting the Article

Ana Lúcia Ribeiro Valadares; Aarão Mendes Pinto-Neto, Lúcia Costa Paiva

Revising It for Intellectual Content

Ana Lúcia Ribeiro Valadares; Aarão Mendes Pinto-Neto; Lúcia Costa Paiva; Maria Helena Sousa; Vanessa SS Machado

Final Approval of the Completed Article

Ana Lúcia Ribeiro Valadares; Aarão Mendes Pinto-Neto; Lúcia Costa Paiva; Maria Helena Sousa; Vanessa SS Machado

b. ~~Conflicts of interest statement~~Competing interests

Formatted: Portuguese (Brazil)

Formatted: Portuguese (Brazil)

Formatted: Portuguese (Brazil)

Formatted: Portuguese (Brazil)

Formatted: Numbered + Level: 1 + Numbering Style: a, b, c, … + Start at: 1 + Alignment: Left + Aligned at: 0.25" + Indent at: 0.5"

For peer review only

As corresponding author, I confirm that I have collected ICMJE Uniform Disclosure Forms for Potential Conflicts of Interest from every author and no Conflicts of Interest exist for any of the authors.

Acknowledgements

c. Financial SupportFunding:

The São Paulo Foundation for the Support of Research (FAPESP), grant # 04/10524-8.

d. Data sharing

We confirm that is no additional unpublished data from the present study available.

Conception and Design

Aarão Mendes Pinto-Neto, Vanessa S S Machado, Ana Lúcia Ribeiro Valadares

Acquisition of Data

Vanessa S S Machado; Aarão Mendes Pinto-Neto

Analysis and Interpretation of Data

Maria Helena Sousa ; Aarão Mendes Pinto-Neto; Ana Lúcia Ribeiro Valadares;

Drafting the Article

Formatted: Numbered + Level: 1 + Numbering Style: a, b, c, … + Start at: 3 + Alignment: Left + Aligned at: 0.25" + Indent at: 0.5"

Formatted: Indent: Left: 0.5"

Formatted: Indent: Left: 0.5"

Formatted: English (U.S.)

Formatted: English (U.S.)

Formatted: English (U.S.)

For peer review only

15

Ana Lúcia Ribeiro Valadares; Aarão Mendes Pinto-Neto, Lúcia Costa Paiva

Revising It for Intellectual Content

Ana Lúcia Ribeiro Valadares; Aarão Mendes Pinto-Neto; Lúcia Costa Paiva; Maria Helena Sousa; Vanessa SS Machado

Formatted: English (U.S.)

Final Approval of the Completed Article

Ana Lúcia Ribeiro Valadares; Aarão Mendes Pinto-Neto; Lúcia Costa Paiva; Maria Helena Sousa; Vanessa SS Machado

Formatted: English (U.S.)

Figure Legend

Figure 1: Age at the onset of diabetes over lifetime (years)

Cumulative survival

N=617

Mean age at onset of the disease was 56 ± 11.2 years (median 55 years)

Cumulative continuation rate without diabetes was 56% at 92 years of age

For peer review only

References

1. Mensing C, Boucher J, Cypress M, et al. National standards for diabetes self-management education. *Diabetes Care* 2005;28 Suppl 1:S72-79.
2. Emerging Risk Factors Collaboration, Seshasai SR, Kaptoge S, Thompson A, et al. Diabetes mellitus, fasting glucose, and risk of cause-specific death. *N Engl J Med* 2011;364:829-841.
3. Whiting DR, Guariguata L, Weil C, Shaw J. IDF diabetes atlas: global estimates of the prevalence of diabetes for 2011 and 2030. *Diabetes Res Clin Pract* 2011;94:311-321.
4. Pi-Sunyer FX. The obesity epidemic: pathophysiology and consequences of obesity. *Obes Res*;10 Suppl 2:97S-104S
5. Falagas ME, Vardakas KZ, Vergidis PI. Under-diagnosis of common chronic diseases: prevalence and impact on human health. *Int J Clin Pract* 2007;61:1569-1579.
6. Bosi PL, Carvalho AM, Contrera D, et al. Prevalence of diabetes and impaired glucose tolerance in the urban population of 30 to 79 years of the city of São Carlos, São Paulo]. *Arq Bras Endocrinol Metabol* 2009;53:726-732.
7. Lebrão ML, Duarte YAO. SABE – Saúde, bem-estar e envelhecimento. O projeto SABE no município de São Paulo: uma abordagem inicial. Brasília (DF): Organização Pan-Americana da Saúde, 2003. Available at: http://www.fsp.usp.br/sabe/Extras/Livro_SABE.pdf. Accessed April 20, 2011.

For peer review only

8. Rosa RS, Schmidt MI. Diabetes mellitus: magnitude das hospitalizações na rede pública do Brasil, 1999-2001. *Epidemiol Serv Saúde* 2008;17:123-153.

9. Köster I, Schubert I, Huppertz E. [Follow up of the CoDiM-Study: cost of diabetes mellitus 2000-2009]. *Dtsch Med Wochenschr* 2012;137:1013-1016.

10. Vargas CM, Burt VL, Gillum RF, Pamuk ER. Validity of self-reported hypertension in the National Health and Nutrition Examination Survey III, 1988-1991. *Prev Med* 1997;26(5 Pt 1):678-685.

11. Snoek FJ. *Management of diabetes: psychological aspects of diabetes management*. Abingdon: The Medicine Publishing Company, 2002.

12. Ministério da Saúde – Brasil. Vigilância de fatores de risco e proteção para doenças crônicas não transmissíveis por entrevistas telefônicas (VIGITEL). Available at: http://portal.saude.gov.br/portal/arquivos/pdf/167_Q2008.pdf. Accessed August 1st, 2011.

13. Guralnik JM, Fried LP, Simonsick EM, Kasper JD, Lafferty ME, eds. *The Women's Health and Aging Study: health and social characteristics of older women with disability*. NIH Pub. No. 95-4009. Bethesda, MD: National Institute on Aging, 1995.

14. Lee ET. Statistical methods for survival data analysis. Belmont. CA: Lifetime Learning Publications, 1980

15. National Diabetes Statistics, 2011. http://diabetes.niddk.nih.gov/dm/pubs/statistics/. Accessed August 1st, 2012.

For peer review only

16. Wennberg P, Rolandsson O, van der A DL, et al. Self-rated health and type 2 diabetes risk in the European Prospective Investigation into Cancer and Nutrition-InterAct study: a case-cohort study. *BMJ Open* 2013;3(3). pii: e002436. doi: 10.1136/bmjopen-2012-002436.
17. Singh-Manoux A, Martikainen P, Ferrie J, Zins M, Marmot M, Goldberg M. What does self rated health measure? Results from the British Whitehall II and French Gazel cohort studies. *J Epidemiol Community Health* 2006;60:364-372.
18. Machado VS, Valadares AL, da Costa-Paiva LS, de Souza MH, Osis MJ, Pinto-Neto AM. Factors associated with self-perception of health among Brazilian women 50 years or older: a population-based study. *Menopause* 2013;20:1055-60.
19. Meisinger C, Kandler U, Ladwig KH. Living alone is associated with an increased risk of type 2 diabetes mellitus in men but not women from the general population: the MONICA/KORA Augsburg Cohort Study. *Psychosom Med* 2009;71:784-788.
20. Lidfeldt J, Nerbrand C, Samsioe G, Agardh CD. Women living alone have an increased risk to develop diabetes, which is explained mainly by lifestyle factors. *Diabetes Care* 2005;28:2531-2536.
21. Schmaltz HN, Southern D, Ghali WA, et al. Living alone, patient sex and mortality after acute myocardial infarction. *J Gen Intern Med* 2007;22:572-578.
22. Agardh E, Allebeck P, Hallqvist J, Moradi T, Sidorchuk A. Type 2 diabetes incidence and socio-economic position: a systematic review and meta- analysis. *Int J Epidemiol* 2011;40:804-818.

For peer review only

23. Stringhini S, Tabak AG, Akbaraly TN, et al. Contribution of modifiable risk factors to social inequalities in type 2 diabetes: prospective Whitehall II cohort study. *BMJ* 2012;345:e5452.

24. Talaei M, Sadeghi M, Marshall T, et al. Anthropometric indices predicting incident type 2 diabetes in an Iranian population: The Isfahan Cohort Study. *Diabetes Metab* 2013 Jul 15. pii: S1262-3636(13)00080-3. doi: 10.1016/j.diabet.2013.04.001.

25. Jeffreys M, Lawlor DA , Galobardes B, McCarron P, Kinra S, Ebrahim S, Smith GD. Lifecourse weight patterns and adult-onset diabetes: the Glasgow Alumni and British Women's Heart and Health studies. *Int J of Obes* 2006; 30:507–512.

26. Kim C, Edelstein SL, Crandall JP, et al., Diabetes Prevention Program Research Group. Menopause and risk of diabetes in the Diabetes Prevention Program. *Menopause* 2011;18:857-868.

27. Morling JR, Balkau B, Wild SH. Diabetes in women - a life-course approach. *Menopause Int* 2013;19:87-95.

28. Rockwood K, Awalt E, MacKnight C, McDowell I. Incidence and outcomes of diabetes mellitus in elderly people: report from the Canadian Study of Health and Aging. *CMAJ* 2000;162:769-772.

29. Bakhshi E, Seifi B, Biglarian A, Mohammad K. Changes in Body Mass Index across Age Groups in Iranian Women: Results from the National Health Survey.J Nutr Metab. 2012;2012:848403.

For peer review only

20

Table 1: Percentage of women without and with diabetes according to their sociodemographic and behavioral characteristics- Bivariate analysis

Variable	N	Without Diabetes %	With Diabetes %	p- value[2]
Age (years)	617			0.04
50 – 59		41.7	30.0	
60 – 69		30.8	36.4	
≥ 70		25.5	33.6	
Schooling (years)	617			0.20
≤ 8		67.9	78.6	
> 8		32.1	21.4	
Marital status[1]	609			0.59
Partner		48.7	45.7	
No partner		51.3	54.3	
Skin color/ethnicity[1]	609			0.76
Other		29.0	30.7	
White		71.0	69.3	
Monthly income (USD)[1]	397			0.07
≤ USD 750		50.8	62.2	
> USD 750		49.2	37.8	
Smoking	617			0.22
Never smoked		62.5	68.5	
Used to smoke		37.5	31.4	
Smoker				
number of cigarettes smoked per day	601			0.92

For peer review only

21

currently or in the past				
≤15		85.4	84.6	
>15		14.6	15.4	
Alcohol consumption	617			0.97
No		85.1	85.0	
Yes		14.9	15.0	
frequency of alcohol consumption	617			0.92
none or < 1 day/month		88.9	88.6	
> 1 day/month		11.1	11.4	
Woman has private medical insurance	617			0.17
No		50.1	57.1	
Yes		49.9	42.9	
Practices physical exercise weekly	617			0.16
No		62.3	69.3	
Yes		37.7	30.7	
Frequency of physical exercise[1]	616			0.74
Up to 2 days/week		73.1	75.0	
≥3 days/week		26.9	25.0	
BMI (kg/m^2)[1]	497			0.007
<25		40.3	25.5	
25 -29.9		36.7	40.0	
≥30		23.0	34.5	
BMI (kg/m^2) at 20-30 years of age[1]	440			0.001
<25		37.2	32.2	
25 -29.9		53.3	44.1	
≥30		9.5	23.7	

For peer review only

Self-rated health	617			0.0001
Other		36.7	56.4	
very good, good		63.3	43.6	
Woman stopped menstruating over one year ago[1]	615			0.95
No		13.4	13.7	
Yes		85.6	85.3	

[1]Some values may not add up to 617 because of missing data.

[2]X^2 test with Yates' correction.

Table 2: Variables associated with the presence of Diabetes– Cox multiple regression analysis (n = 428)

Variable	Coefficient	Standard error of the coefficient	P-value
Self-rated health (very good, good)	-0.792	0.215	<0.001
Number of individuals living in the household (> 2)	0.656	0.223	0.003
BMI (kg/m^2) at 20–30 years of age	0.056	0.023	0.014

For peer review only

Predictor variables taken into consideration: schooling (≤ 8 years: 0 / > 8 years: 1); marital status (with a partner: 1 / no partner: 0); skin color (white: 1 / other: 0); number of people living in the household (≤ 2: 0 / > 2: 1); smoking (never smoked: 0 / past or current smoker: 1); number of cigarettes smoked per day currently or in the past (≤ 15: 0 / > 15: 1); alcohol consumption (yes: 1 / no: 0); frequency of alcohol consumption (none or < 1 day/month: 0 / other: 1); woman has private medical insurance (yes: 1 / no: 0); practices physical exercise weekly (yes: 1 / no: 0); frequency of physical exercise (≤ 2 days/week: 0 / ≥ 3 days/week: 1); BMI (kg/m^2) at 20-30 years of age; self-rated health (very good, good: 1 / other: 0); menopausal (yes: 1; no: 0).

For peer review only

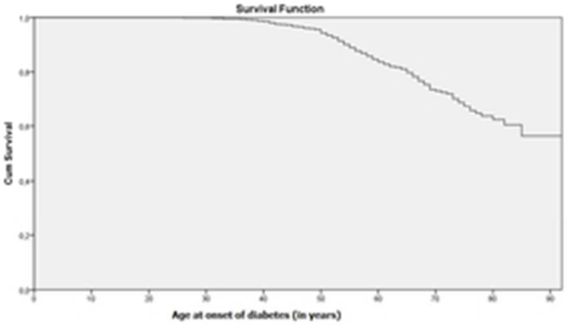

14x7mm (600 x 600 DPI)

peer review only
